# Editorial: The role of muscle secretome in health and disease—Volume II

**DOI:** 10.3389/fphys.2023.1167063

**Published:** 2023-02-27

**Authors:** Céline Aguer, Domenico Di Raimondo

**Affiliations:** ^1^ Department of Physiology, Faculty of Medicine and Health Sciences, McGill University—Campus Outaouais, Gatineau, QC, Canada; ^2^ Institut du Savoir Montfort, Ottawa, ON, Canada; ^3^ Department of Biochemistry, Microbiology and Immunology, Faculty of Medicine, University of Ottawa, Ottawa, ON, Canada; ^4^ School of Human Kinetics, Faculty of Health Sciences, University of Ottawa, Ottawa, ON, Canada; ^5^ Interdisciplinary School of Health Sciences, Faculty of Health Sciences, University of Ottawa, Ottawa, ON, Canada; ^6^ Department of Promoting Health, Maternal-Infant, Excellence and Internal and Specialized Medicine “G. D’Alessandro” (PROMISE), University of Palermo, Palermo, Italy; ^7^ PhD Program Molecular and Clinical Medicine, Department of Promoting Health, Maternal-Infant, Excellence and Internal and Specialized Medicine “G. D’Alessandro” (PROMISE), School of Medicine, University of Palermo, Palermo, Italy

**Keywords:** myokines, exercise, muscle secretome, extracellular vesicles (EVs), muscle derived metabolites

In the first edition of our Research Topic, we have tried to collect a series of articles, including reviews and original research, that could represent as reliably as possible the growing interest in muscle secretome among researchers worldwide. Of the 12 articles that we collected at the end of the first edition, no less than ten analyzed some novelties related to myokines, which are among the main actors allowing the muscle to communicate with distant organs, partly controlling its metabolism and biological functions in both health and disease. The relationship that exists between the production and/or release of myokines from resting muscle and muscle contraction is variable: it may depend on the level and type of physical activity performed or may not be related to it at all. In the editorial that accompanied the first edition (Aguer et al.), we attempted to draw an identikit including the essential characteristics needed for a molecule to be identified as a myokine. This shows how we are still moving in an extremely fluid and constantly updated field of research, which almost every month provides us with news and opens new scenarios that were not conceivable only a short time before.

The muscle secretome contains thousands of compounds of which myokines are probably only a small part. There has been accumulating evidence for exercise-induced skeletal muscle secretion of microRNAs, mitochondrial DNA, various metabolites, and enzymes. The role of exosomes and extracellular vesicles (EVs) in the transport of some of these factors has also been in the spotlight over the past 5 years and was the subject of two excellent reviews in Vol 1 of our Research Topic.

With this second volume, we are broadening the discussion by adding new Review Articles and Original Papers addressing the role of the muscle secretome in health and disease.

Bringing our editorial experience to a close, enriched by confrontation with the hundreds of possible authors we have contacted over the years and by the guidance and editorial support from our friends at Frontiers in Physiology, whom we continue to thank for their assistance, we present the four original contributions that are included in the Vol II, which further add new insights to the large, multifaceted and fascinating picture of the muscle secretome.

The original research by Zakharova et al. investigated a very interesting topic: They studied whether the time of day when training is performed differently modify the content of cytokines in mice skeletal muscles with metabolic disorders induced by a 16-week high fat diet (HFD). HFD affected muscle myokine levels: the content of interleukin 6 (IL-6), IL-15, and chemokine ligand 1 (CXCL1) decreased. The treadmill training caused multidirectional changes in the concentration of myokines in muscle tissue in all groups of animals tested, mainly on IL-6 levels, but also on IL-15. The changes depended to the greatest extent on the training time scheme, demonstrating the potential of training to positively influence the alterations induced by metabolic disorders.

The review by Leuchtmann et al. addressed the issue of the analysis of the secretome of long-term training adaptation. The changes induced by regular exercise on the muscle secretome depend on the type of exercise performed, endurance or resistance. The contribution of non-myokine factors, including metabolites, enzymes, microRNAs, or mitochondrial DNA transported in extracellular vesicles, also appears to be relevant. By analysing in depth the many molecules that may become a target for researchers, the review highlights not only their role in mediating increased performance in athletes but also their potential significance as targets in numerous clinical syndromes.

The review article by Darragh et al. draws attention to the extracellular vesicles (EVs), and in particular, a sub-category termed “small EVs.”, which are the carriers through which many of the molecules that are produced during exercise are delivered into the circulation.

Finally, the fourth review by Ahsan et al. debates the ability of myokines to influence energy metabolism *via* AMP-activated protein kinase (AMPK) signalling. AMPK is a key system centrally involved in the regulation of glucose and lipid metabolism. The review highlights that AMPK is not only a mediator of the positive role certain myokines have on muscle glucose and fatty acid metabolism, but also that the secretion of some of these myokines is regulated in an AMPK-dependent manner, showing there may be a feedback loop between myokines and AMPK in the regulation of muscle metabolism.

These articles expand the understanding of the muscle secretome, which is constantly evolving from being almost synonymous with myokines to encompassing a much broader catalogue of molecules. We can consider the goal we set ourselves a few years ago in accepting the assignment to renew our Research Topic for a second edition to have been achieved: to keep up interest in a field of research with enormous potential by approaching it both from the inside, i.e., by analysing its bio-molecular aspects, and from the outside, i.e., from the point of view of the athlete, who expects indications as to how they can improve their physical performance in a healthy and safe manner, and from the point of view of the patient, who can reasonably hope to receive from this new researches therapeutic perspectives for the many pathologies that (regardless of the organ initially affected) compromise the functional capacities of the muscle.

